# A systematic review of studies with a representative sample of refugees and asylum seekers living in the community for participation in mental health research

**DOI:** 10.1186/s12874-017-0312-x

**Published:** 2017-03-02

**Authors:** Joanne C. Enticott, Frances Shawyer, Shiva Vasi, Kimberly Buck, I-Hao Cheng, Grant Russell, Ritsuko Kakuma, Harry Minas, Graham Meadows

**Affiliations:** 1 Department of Psychiatry, Southern Synergy, Monash University, 126-128 Cleeland St, Dandenong, VIC 3175 Australia; 2 RDNS Institute, 31 Alma Rd, St Kilda, VIC 3182 Australia; 3 Southern Academic Primary Care Research Unit, School of Primary Health Care, Monash University, Building 1, 270 Ferntree Gully Road, Notting Hill, VIC 3168 Australia; 4 Global and Cultural Mental Health Unit, Centre for Mental Health, Melbourne School of Global and Population Health, The University of Melbourne, Level 4, 207 Bouverie Street, Parkville, Victoria 3010 Australia; 5 Mental Health Program, Monash Health, Dandenong, Victoria 3075 Australia

**Keywords:** Refugee, Asylum seeker, Stateless person, Mental disorders, Hard-to-reach, Hidden population, Sampling, Recruitment, Surveying

## Abstract

**Background:**

The aim was to review the literature to identify the most effective methods for creating a representative sample of refugee and asylum seeker groups living in the community to participate in health and mental health survey research.

**Methods:**

A systematic search of academic and grey literature was conducted for relevant literature with ‘hidden’ groups published between January 1995 and January 2016. The main search used Medline, PsycINFO, EMBASE, CINAHL and SCOPUS electronic databases. Hidden groups were defined as refugees, asylum seekers, stateless persons or hard/difficult to reach populations. A supplementary grey literature search was conducted. Identified articles were rated according to a created graded system of ‘level of evidence for a community representative sample’ based on key study factors that indicated possible sources of selection bias. Articles were included if they were assessed as having medium or higher evidence for a representative sample. All full-text papers that met the eligibility criteria were examined in detail and relevant data extracted.

**Results:**

The searches identified a total of 20 publications for inclusion: 16 peer-reviewed publications and four highly relevant reports. Seventeen studies had sampled refugee and asylum seekers and three other hidden groups. The main search identified 12 (60.0%) and the grey search identified another eight (40.0%) articles. All 20 described sampling techniques for accessing hidden groups for participation in health-related research. Key design considerations were: an a priori aim to recruit a representative sample; a reliable sampling frame; recording of response rates; implementation of long recruitment periods; using multiple non-probability sampling methods; and, if possible, including a probability sampling component. Online social networking sites were used by one study. Engagement with the refugee and asylum seeker group was universally endorsed in the literature as necessary and a variety of additional efforts to do this were reported.

**Conclusions:**

The strategies for increasing the likelihood of a representative sample of this hidden group were identified and will assist researchers when doing future research with refugee groups. These findings encourage more rigorous reporting of future studies so that the representativeness of samples of these groups in research can be more readily assessed.

**Electronic supplementary material:**

The online version of this article (doi:10.1186/s12874-017-0312-x) contains supplementary material, which is available to authorized users.

## Background

Population health surveys are typically used to determine health status, healthcare needs and health service utilization patterns in particular populations. However, despite efforts to ensure findings are representative of the population of interest, certain “hidden” groups are inevitably excluded - people of refugee or asylum seeker (RAS) backgrounds being one such group [1–5]. Hidden groups present special challenges for the sample process including representative sampling and coverage, identification, contact, recruitment, and data collection [1–10]. Mental health issues are a problem for RAS populations worldwide [11, 12] and this can compound sampling challenges. There are likely to be issues around trust and safety, particularly for those with a history of torture and trauma, as well as concerns about stigma and privacy – especially if refugee status is undetermined. Low rates of health literacy in some refugees may also impact on the willingness and capacity to be involved in research and disclose information related to mental health. There is little high quality published evidence about mental health and health services use among RAS groups [13, 14]. Understanding the mental health needs of RAS groups living in the community, especially within communities with large RAS populations, is needed to inform service delivery to this vulnerable group [15].

Representative samples are subgroups of people that contain all the elements of interest from a target population [5]. The sample frame represents a list of the target population from which the sample is selected, and ideally contains all elements in the target population. Sometimes the frame can consist of the entire target population, but this is uncommon. The sampling frame should be clearly defined and have measurable characteristics before a representative subgroup is sought. Gender representative samples, for instance, will endeavour to match the proportion of each gender as in the target population. The representativeness of the sample depends on the quality of the sampling frame [16]. The lack of a sampling frame or rapidly changing frames for many RAS groups is a known barrier to conducting research with RAS populations [1–6].

By definition, a reliable frame and a representative sampling mechanism cannot be easily established for hidden groups [17]. Inherent problems within these two important aspects of sampling are sources of selection bias [17]. Even if the relevant frame is understood, for example by using a host country immigration records, methods to select RAS participants are often not conducive to representative sampling. Sampling techniques commonly used are those that promote participation in known RAS individuals such as convenience sampling, e.g., research participants selected because they are known to the researchers, or snowballing, e.g., research participants are sought through chain-referral by other research participants [2, 10]. Generally convenience and snowball samples are non-representative because the sampling coverage is limited to the contact circles of certain people and are thus subject to selection bias [10, 17].

This systematic review aimed to identify methods to achieve representative samples of RAS groups living in the community for participation in mental health survey research. However, since methods could be transferred from research seeking representative samples from other at risk groups that are characterized as hidden such as men who have sex with men [18, 19], this review also includes health-related research involving participants from other hidden groups, but only if the sampling methods were suggested as potentially transferable to other groups. Motivation for individuals to participate in health research can be different if a service is offered in exchange for participation [20]; for example, survey research on oral health behaviours that incentivizes participation using a free dental examination is likely to recruit individuals who want a dental examination. Whereas research involving only surveys about dental hygiene, without a service in exchange, might not attract the same participants. This systematic review concentrated on the latter strategy. The question that we aimed to answer through this systematic review was, “*What are the most effective methods for creating a representative sample of RAS living in the community, to participate in health and mental health survey research?*”

## Methods

We followed the PRISMA (Preferred Reporting Items for Systematic reviews and Meta-Analyses) statement for conducting and reporting a systematic review [17, 21]. A systematic search of both academic and grey literature identified available studies that met the inclusion criteria. To ensure a comprehensive representation of the literature, we included papers that used qualitative, quantitative, mixed methods and case study methodologies, and cross-sectional, cohort, experimental and observational designs. We did not restrict the search to population surveys because these studies are resource-heavy and infrequently conducted within hidden groups. The review processes are given below. Also see the section describing author contributions for further details of who undertook the review tasks.

### Main search strategy – peer-reviewed literature

Searches of the Medline, PsycINFO, EMBASE, CINAHL and SCOPUS electronic databases were conducted for English language papers published between January 1995 and January 2016. The following medical subject headings and keywords were sought: [“refugee*” OR “asylum seeker*” OR “stateless person*” OR “difficult to reach” OR “hard-to-reach” OR “hidden population*”] AND [“sampling”, “recruitment” OR “surveying” OR “sampling studies”]. Note the *asterisk indicated a word that was truncated during the search. The search purposely included papers not indexed with a RAS term, in case relevant papers had included this information elsewhere and was inclusive of all age groups, not restricted to ‘adults’ because this may have excluded papers that did not specify the age group of their sample. In addition, manual checks of the reference lists of retrieved papers and citation searches were conducted.

The initial searches were performed in January 2015 and subsequently re-run in PubMed and PsycINFO in January 2016 to identify additional relevant studies published in 2015. No additional studies were identified.

### Initial inclusion criteria

To pass an initial screen, abstracts and titles needed to contain enough information to indicate that the study had focussed on *health research* and referred to methods of recruitment for *hidden groups*. Hidden groups were defined as refugees, asylum seekers, stateless persons or hard/difficult to reach populations such as men who have sex with men [18, 19].

Full text articles were retrieved for all records that passed the screen, or if exclusion could not be determined during the screen. Full text articles were then examined and met the eligibility for analysis criteria if they were:

1. Published in the English language
2. Peer-review publication (this condition was waived for the grey literature search)
3. Primary article providing original data
4. Focus on health and a community sample (not in-patient, etc.)
5. Focus on adults
6. Published January 1995 – January 2016
7. Study sample frame is a hidden population
8. Sample of RAS or another hidden sample with methods described to be potentially transferable to RAS
9. Reports the sampling technique in sufficient detail to replicate sampling
10. Focus on original data collection (not census, hospital administrative data etc.)

Articles were excluded if they were classified as an incomplete article (e.g., conference abstract, editorial, commentary or letter); offered a service to study participants; reported data already used in another included article; or were review articles.

### Final inclusion criteria – and study quality assessment

Full text articles that met the initial inclusion criteria then underwent an assessment for study quality which consisted of an analysis for level of evidence for obtaining a representative sample. The final inclusion criteria required studies to have achieved a *medium-high* level evidence of obtaining a representative sample. This quality assessment process is described immediately below and summarised in Table 1.

**Table 1:** Level of evidence for a representative sample (high, med, low, unclear)

High	‘High’ level of evidence for a representative sample *(or ‘low risk’ of selection bias).*	1.The investigators describe a clear, defined and reliable sample frame for the target group •Sample frame already known e.g. hidden group reliably detected in census data or registry •Sample frame created by the researchers and includes the vast majority of the hidden target group 2.The investigators describe a random component in the process of drawing from the sample frame such as: •Referring to a random number table; •Using a computer random number generator *Note: Must fulfil both 1 and 2 criterion for a judgement to be made of ‘high’ level evidence for representativeness.
Medium	‘Medium’ level of evidence for a representative sample *(or ‘medium risk’ of selection bias*)	Sampling frame and sampling processes are applied from both the high and low below criteria. Non-random sampling with •Multiple efforts and techniques used with the a priori aim to approximate a representative sample including two or more of: oRespondent driven sampling (RDS) oQuota sampling oMaximum variation oMultiple starting points for snowballing oSample representativeness ascertained e.g. sample compared with census demographics of the hidden group.
Low	‘Low’ level of evidence for a representative sample *(or ‘high risk’ of selection bias*)	1.The investigators do not use a comprehensive sample frame for the hidden target group •Sample frame likely to exclude a significant proportion of the target group 2.The investigators describe a non-random component in the sampling process, for example: •Snowballing •Convenience sampling 3.Low sample numbers 4.Low response rates
Unclear	‘Unclear’ evidence for a representative sample *(or unclear selection bias*).	Indicates a lack of information about the sample frame and sample drawn.

Non-representative community sampling (or selection bias) due to inadequate generation of a randomized sample from a reliable sample frame was assessed using the above judgement criteria

Determining the level of evidence for a representative sample involved a quality assessment of each study for potential sources of bias. This assessment was performed by two authors (JE and KB) and differences were resolved through discussion and consensus. Sources of selection bias can particularly compromise the establishment of a representative sample [17]. Other sources of bias exist, but the key obstacles to initially overcome are those related to selection bias. Our judgement criteria for assessing selection bias was adapted from Higgins and Green [17]. We produced a graded system of ‘level of evidence for a community representative sample’ based on key study factors that indicated possible sources of selection bias [17]. Table 1 outlines this graded system.

### Grey literature search strategy

The novel, and we think unique, grey literature search parameters described below were devised from input from an expert advisory committee that included senior researchers experienced in the sourcing of refugee publications.

The systematic search of grey literature centred on 23 countries: Australia, Canada and United States of America plus the top 20 countries in the UNHCR global rankings of highest refugee “third country resettlement” intake per 1000 inhabitants in 2010 [22], (see Additional file 1: Appendix A). For each of these 23 countries, a maximum of 2 hours per country was allocated to search the Internet for relevant literature. Instructions given to the research assistants who performed this task were to: identify the website for the Department of Health in that country and spend a maximum of one hour searching this website using the same search terms as in the main search; and next, identify any website for the statistical department in that country and again spend a maximum of 1 hour searching this website for relevant articles.

This search was supplemented with a general Internet search using Google and Google Scholar. In addition, we asked the investigators to identify relevant sources of literature that could be in the form of: websites, newsletters (online or print), reports (online or print), annual reports, research or quality assurance reports, any persons that had sampled hidden refugee and asylum seeker populations, and any another relevant contact person. Free text searching was implemented using the same search terms as in the main search. Grey literature were subjected to the same inclusion criteria described above.

### Data collection

Papers identified as eligible for analysis were read and key information extracted by research assistants. This included the study focus (mental health or health), design, study setting, target sample size and descriptors of the study’s target population. Other extracted data consisted of information from all stages of a research study where representativeness may be threatened: key sources of potential selection bias such as the development or defining of a sampling frame; random (or non-random) selection components; recruitment and sampling methods/considerations/techniques; the barriers to participation in health research; strategies implemented to improve participation; and response rates and attrition.

Only findings reported in the original publications or publications using the same study data were used for extraction. Authors were not contacted for additional information.

Meetings with the research assistants occurred regularly, and any discordance during the search, extraction and assessment tasks was resolved by a consensus panel, which included the research assistant(s) and two senior authors (FS & JE).

## Results

**Fig. 1:**
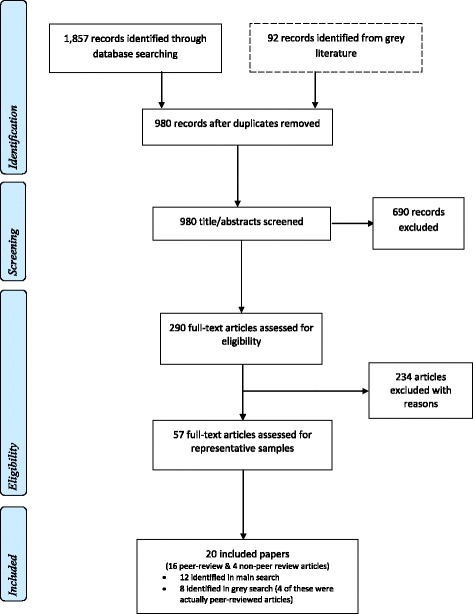
Flow Diagram of combined main and grey search strategies to identify eligible papers. For further details about the grey search, see Additional file 1: Appendix A and Additional file 3: Appendix C, and the main search, see Additional file 2: Appendix B

The searches identified a total of 20 publications for inclusion. A summary of the search strategy is shown in Fig. 1, and details of the separate main and grey searches are available in Additional files 2 and 3: Appendices B and C, respectively. As per the eligibility criteria, all 20 had achieved a community representative sample of a hidden group, where the level of evidence for a representative sample had been rated by the reviewers as medium or higher [18, 19, 23–40]. Seventeen had specifically sampled RAS and three involved another hidden group [18, 19, 38]; see Table 2. All 20 publications described sampling techniques for accessing hidden groups for participation in health-related research (see Table 2).

**Table 2:** Publications describing studies included in this review (*n* = 20**)**

Author(s)	Country	Study design & focus	Sample frame: Type of ‘hidden’ population residing in the community	N and response rates
Fenta et al. 2006 [32]	Canada	Cross-sectional, mental health	Ethiopian immigrants/refugees	*n* = 340, response rate 85%.
Silove et al. 2007 [39]	Australia	Cross-sectional, mental health	Vietnamese refugees who have been in Australia for 10+ years	*n* = 1,161, response rate 82%
**De Maio et al. 2014** [30] ^**G**^	**Australia**	**Longitudinal, mental health**	**Refugees granted residency within previous 3–6 months**	***n*** **= 2,400, response rate approximately 60%**
**McAuliffe 2013** [37] ^**G-Report**^	**Australia**	**Cross-sectional, health (or applicable to health)**	**Irregular maritime arrivals to Australia issued with a protection visa within specified timeframe**	***n*** **= 1,008, response rate 47%**
**Commissariat for Refugees 2008** [29] ^**G-Report**^	**Serbia**	**Cross-sectional, mental health**	**Refugees, predominantly from former Yugoslavia and Croatia**	***n*** **= 3,684, response rate not reported**
**Citizenship and Immigration Canada 2011** [28] ^**G-Report**^	**Canada**	**Cross-sectional, mental health**	**Refugees (Afghan 22%)**	***n*** **= 501, response rate 41%.**
Cochran et al. 2013^G^/Ao (2016) [23]*	USA	Cross-sectional, mental health	Bhutanese refugees	*n* = 579, response rate 73%
Maximova & Krahn 2010 [36] ^G^	Canada	Cross-sectional, mental health	Refugees (63% Yugoslavian)	*n* = 525, overall response rate (in parent study) 95%
Gerritsen et al. 2006 [33]	The Netherlands	Cross-sectional, mental health	Refugees & Asylum seekers	*n* = 178, response rate (for refugees) 59%
Spring et al. 2003 [40]	USA	Multiphase epidemiologic study, torture prevalence	Somalian and Oromo refugees	*n* = 1,165, response rate 97.1%.
Bhui et al. 2006 [24]	UK	Mix-method, mental health	Somalian refugees	*n* = 143, response rates 76%–83%
Bilsborrow et al. 2011 [25]	USA	Cross-sectional, wellbeing	Colombian migrants (including asylum seekers) in Ecuador	*n* = 234 households, response rate 76%
Blight et al. 2006 [26]	Sweden	Cross-sectional, mental health	Refugees from Bosnia-Herzegovina	*n* = 413, response rate 63.5%.
Heeren et al. 2012 [34]	Switzerland	Cross-sectional, mental health	Asylum seekers who arrived less than 2 years ago in Zurich	*n* = 126, response rate 68.3%
Khavarpour & Rissel 1997 [35]	Australia	Cross-sectional, mental health	Iranian migrants and refugees	*n* = 413, response rate 99% (phone) *n* = 161 (follow-on postal survey)
Qiu et al. 2012 [38]	China	Cross-sectional, investigating sampling & applicable to health research	Migrants in China	*n* = 1,270, response rate not reported
Vial et al. 2014 [18]	USA	Cross-sectional, health	Men who have sex with men	*n* = 3,640, response rate not reported
Wylie & Jolly 2013 [19]	Canada	Cross-sectional, health & investigating sampling	Men who have sex with men and sex workers	*n* = 578, response rate not reported
Bogic et al. 2012 [27] ^G^	Germany	Cross-sectional, mental health	Refugees from former Yugoslavia	*n* = 854, response rate 52.9%
Dunlavy 2001 [31] ^G-Thesis^	Sweden	Cross-sectional, mental health	African refugees and immigrants	*n* = 420, response rate not reported

The studies are listed based on the ranking for a representative sample: *high* at the top and *medium* at the bottom (for the specific assigned ranks, see Table 3). This table includes 17 studies focusing on refugees and asylum seekers and 3 studies focusing on another hidden group. Non-peer-reviewed publications are emphasize in bold in table. *Note that Cochran et al. [41] is a non-peer reviewed article that was identified during the grey literature search, which lead to the peer-reviewed publication by Ao et al. [23] which describes the same study

^G^ Identified in grey literature search. ^G-Report^ Government reports identified in grey literature search. ^G-Thesis^ Dissertations identified in grey literature search

### Main search and separate grey search

In the main search, 1857 records were initially identified in electronic databases and another 29 were recovered from reference checks, see Fig. 1 and Additional file 2: Appendix B. After removing duplicates, 893 records underwent abstract/title screening, of which 238 progressed to full-text examination for eligibility. Reasons for failing the full-text examination are indicated in Additional file 2 Appendix B, and only 36 progressed to undergo assessment by the reviewers for having a representative sample(s). A further 24 were excluded because these studies failed the criteria for having medium to high level evidence of representative samples. Therefore, there were 12 included publications identified in the main search.

In the grey search, 92 records were initially identified and underwent abstract/title screening, of which 52 progressed to full-text examination for eligibility (see Additional file 3: Appendix C). Reasons for failing the full-text examination are indicated in Additional file 3: Appendix C, and 21 progressed to undergo assessment by the reviewers for a representative sample(s). A further 13 were excluded because these studies failed the criteria for having medium-high level evidence of representative samples. Therefore, the grey search identified eight publications.

Of the 20 publications included in this review, the main search identified 12 papers (60.0%) and the grey literature search identified another eight papers (40.0%). Half of the publications identified from the grey search were peer-reviewed publications (4/8, 50.0%), see Tables 2 and 3. Overall, this review includes 16 peer-reviewed publications [18, 19, 23–27, 31, 34, 36, 38–40], one non-peer reviewed protocol report [30], and three governmental reports [28, 29, 37]; see Table 2. The latter four reports were identified during the grey literature search.

**Table 3:** Additional details of the studies included in this review (*n* = 20)

Author(s)	Community representative sample*	RAS	Sampling method(s)	Sampling techniques					Sampling considerations		
									*a priori* aim	Used registry/census data	
				Multiple non-probability methods	Probability (random) component	Network-based				In sampling/recruitment	In assessing representativeness
						Snowballing	RDS	Online			
Fenta et al. 2006 [32]	high	Y	Random sampling from created frame	✗	✓	✓	✗	✗	✗	✓ Lists from Ethiopian organizations, telephone directory	✗
Silove et al. 2007 [39]	high	Y	Probabilistic sampling from created frame (house-to-house screening)	✗	✓	✗	✗	✗	✓	✓	✓
**De Maio et al. 2014** [30] ^**G**^	**high**	**Y**	**All eligible refugees listed in government settlement database were invited**	✗	**All in sample frame invited to participate**	✗	✗	✗	✓	✓	✓
**McAuliffe 2013** [37] ^**G-Report**^	**high**	**Y**	**Sample frame of eligible refugees listed in government settlement database. Quota sampling also used**	✗	✗	✗	✗	✗	✓	✓	✓
**Commissariat for Refugees 2008** [29] ^**G-Report**^	**med/high**	**Y**	**Multistage stratified sampling**	✗	**Unclear**	✗	✗	✗	✗	✓**Municipality registries**	✓
**Citizenship and Immigration Canada 2011** [28] ^**G-Report**^	**med/high**	**Y**	**Random sampling from created frame**	✗	✓	✗	✗	✗	✓	✓ **Government database**	✓
Cochran et al. 2013^G^/Ao (2016) [23]*	med/high	Y	State-based stratification with random sampling from created frame. Supplementary purposive and probability proportional to size sampling	✓	✓	✗	✗	✗	✓	✓	✓
Maximova & Krahn 2010 [36] ^G^	med/high	Y	Systematic sampling (every n^th^ name) from sampling frame	✗	Systematic every n^th^ name in database	✗	✗	✗	✓	✓	✓ Government settlement database
Gerritsen et al. 2006 [33]	med/high	Y	Random samples of refugees were obtained from population registries, plus asylum seekers living in randomly selected reception centres	✗	✓	✗	✗	✗	✓	✓	✗
Spring et al. 2003 [40]	med/high	Y	Multiple purposive sampling methods: Targeted, convenience, snowball sampling	✓	✗	✓	✗	✗	✓	✗	✓ School enrolments, birth statistics, state resettlement records
Bhui et al. 2006 [24]	medium	Y	Community based sampling (convenience) & primary care registry lists (random)	✗	✓	✗	✗	✗	✗	✓	✗
Bilsborrow et al. 2011 [25]	medium	Y	Oversampling (probability sampling) first with supplementary snowball sampling	✗	✓	✓	✗	✗	✗	✓	✗
Blight et al. 2006 [26]	medium	Y	Random sample drawn from a large registry of community living target group	✗	✓	✗	✗	✗	✗	✓	✗
Heeren et al. 2012 [34]	medium	Y	National register of adult asylum seekers (sampled consecutively)	✗	✗	✗	✗	✗	✗	✓	✗
Khavarpour & Rissel 1997 [35]	medium	Y	Snowball sampling with strategies to access diverse social networks	✗	✗	✓	✗	✗	✓	✓	✓
Qiu et al. 2012 [38]	medium	N	Respondent driven sampling	✗	✗	✗	✓	✗	✓	✗	✓ Gender ratio
Vial et al. 2014 [18]	medium	N	Field (convenience) and online sampling	✓	✗	✗	✗	✓	✗	✗	✗
Wylie & Jolly 2013 [19]	medium	N	Respondent driven sampling	✗	✗	✗	✓	✗	✓	✗	✗
Bogic et al. 2012 [27] ^G^	medium	Y	Multiple random and non-random sampling: resident registers, snowballing, community-based sampling	✓	✓	✓	✗	✗	✗	✓	✗
Dunlavy 2001 [31] ^G-Thesis^	medium	Y	Non-probability stratified quota sampling, community-based snowballing with multiple starting points	✓	✗	✓	✗	✗	✓	✓	✗

RAS = refugee and/or asylum seeker participants. Non-peer-reviewed publications are emphasize in bold in table

^G^ Identified in grey literature search. ^G-Report^ Government reports identified in grey literature search. ^G-Thesis^ Dissertations identified in grey literature search

The peer-reviewed publication by Ao et al. [23] is shown together with the non-peer reviewed publication by Cochran et al. [41] in Table 2, because the former was only identified after the latter publication was found during the grey search; both describe the same study and Ao et al. [23] was not identified in the main search.

### Evidence level of a community representative sample

Only four studies (20.0%, 4/20) were rated as having ‘high’ level of evidence for achieving a community representative sample for a hidden group; therefore these four studies were judged to have ‘low risk’ of selection bias, see Tables 1 and 3. These high quality representative samples were from a Canadian study consisting of 340 Ethiopian refugee migrants [32], and three large-scale Australian studies with RAS groups [30, 37, 39]. Two of these four studies were identified in the grey search only and are non-peer reviewed articles [30, 37].

Another six studies with refugees were graded as having medium-high evidence of a representative sample [23, 28, 29, 33, 36, 40] and the remaining ten studies were rated as demonstrating medium evidence; seven of these had sampled RAS [24–27, 31, 34, 35] and the remaining three had sampled other hidden groups [18, 19, 38].

### Sampling techniques

Probability (random) sampling procedures were used in 50.0% (10/20) of studies [23–28, 32, 33, 39]; see Table 3. Three studies did not use any random strategies but instead attempted to invite all eligible participants from within the defined sample frame; two of these were rated high for a representative sample and were large studies involving Australian refugees [30, 37]; and the third rated medium was a Swiss study that had sampled consecutively from a national register of adult asylum seekers [34]. Another study used systematic sampling of every n^th^ name from a sampling frame [36], which is not strictly ‘random’ sampling. Yet another study applied multistage stratified and quota sampling, but because the authors did not specify if there was a random component in the sampling, it remains undetermined whether probability method(s) were employed [29]. The remaining six studies used only non-probability sampling methods [18, 19, 31, 35, 38, 40].

Networked-based sampling techniques were described in nine (45.0%) of the 20 reviewed studies; six included snowballing (30.0%) [25, 27, 31, 32, 35, 40], four purposive/convenience sampling (20.0%) [18, 23, 24, 40], two used respondent driven sampling (RDS; 10.0%) [19, 38], and one utilized online sampling via social media platforms such as Facebook [18]. Four studies applied some type of probability (random) sampling methods and supplemented this with non-probability sampling, such as snowballing or convenience sampling [23–25, 27]; see Table 3.

### Other design issues

Table 3 summarizes the sampling frame, sampling methods and other sampling considerations reported in the 20 included studies. More than half of the studies (65.0%, 13/20) expressed an a priori aim to approximate a representative sample [19, 23, 28, 30, 31, 33, 35–40]. Table 4 summarizes further information on the 20 studies focusing on possible barriers and identified threats to representative samples. Long recruitment periods of between 12 and 25 months were noted to facilitate recruitment from hidden groups in four studies [24, 27, 32, 40]. Weighting methods can be used to adjust the obtained sample to be representative of the target population, and were reported in five studies [25, 30, 37–39].

**Table 4:** Barriers and other factors impacting the achievement of a representative sample (*n* = 20)

Author(s)	Long recruitment(>12 months)	Engagement with ‘hidden’ group	Barriers noted
Fenta et al. 2006 [32]	✓ 12 months	Field-workers spoke the target language	Difficult to identify Ethiopian Muslim names in the telephone directory Potential candidates may have been excluded if they had no telephone, stable address or membership in the Ethiopian organizations used to develop the sample frame
Silove et al. 2007 [39]	✗	Field-workers spoke the target language	Sampling strategy favoured Vietnamese refugees living in ethnically dense areas
**De Maio et al. 2014** [30] ^**G**^	✗	**Community consultation during development of design and methodology Community Engagement officers (members of local migrant communities) recruited to advocate for study, assist with recruitment etc.** **Field-workers spoke the target language** **Interviews conducted in respondent’s homes**	**The high mobility of the target sample made obtaining accurate contact information challenging**
**McAuliffe 2013** [37] ^**G-Report**^	✗	**Bilingual assistants available to assist with survey administration**	**Participants in initial sample excluded if lived in non-metro areas of target cities, lacked a valid phone number or encountered significant language barriers**
**Commissariat for Refugees 2008** [29] ^**G-Report**^	✗		**Contact details of refugees living in private accommodation not all available/correct in municipality records – highly mobile refugees may have been excluded. Substitutions identified by “trustees” – no explanation of how these selections were made**
**Citizenship and Immigration Canada 2011** [28] ^G-Report^	✗	**Promotional materials (posters, FAQ brochures) distributed to service provider organizations to encourage eligible participants to respond**	**Consent given through the returning of a postal questionnaire. Possible self-selection bias (e.g. higher proportion of university education). Poor health or mental health could have been associated with non-response**
Cochran et al. 2013^G^/Ao (2016) [23]*	Not reported	Field-workers spoke the target language Interviews conducted in respondent’s homes	Lack of contact information for eligible participants
Maximova & Krahn 2010 [36] ^G^	✗		Refugees without available addresses in the government database were excluded, as were those who had relocated from study site
Gerritsen et al. 2006 [33]	✗	Field-workers spoke the target language Potential respondents contacted by letter and in person	Recruitment only conducted in municipalities that agreed to provide researchers with contact details of potential participants One third of potential participants had incorrect contact details or were absent when interviewers visited
Spring et al. 2003 [40]	✓ 25 months	Field-workers spoke the target language. Interviews conducted in respondent’s homes. Field staff maintained a presence in the communities, including after hours and weekends. Created marketing materials (e.g. posters) and recruited at many varied community events and locations	Limited to one person per household. Analyses indicated some significant differences on outcome variables depending on recruitment strategy
Bhui et al. 2006 [24]	✓ 12 months	Researchers of same ethnicity as target population networking with local stakeholders to gain trust. Data collection also at weekends and evenings	Census data in the UK does not include country of origin. Authors note that this makes establishing a reliable sampling frame difficult. It was also noted that research fatigue and a failure to see immediate benefits to health and social status were additional barriers to participating in research
Bilsborrow et al. 2011 [25]	✗		Use of archival census data could not identify recent or highly mobile refugees/migrants, or those living in the country illegally
Blight et al. 2006 [26]	✗	Attempts made to reduce focus on ethnicity in the questionnaire & cover letter to account for refugees who no longer identify as refugees	Consent given through the returning of the postal questionnaire. Poor health or mental health (such as concentration difficulties) could have resulted in non-completion.
Heeren et al. 2012 [34]	✗		Reasons for non-participation included lack of time, indifference, distrust of researchers. Authors noted that RAS may feel intimidated or fearful of the interview situation, which may remind them of interviews or interrogations with officials in their home country
Khavarpour & Rissel 1997 [35]	✗	Field-workers spoke the target language	The mailed survey component of the study required participants to supply a postal address. This loss of anonymity was a noted barrier to participation
Qiu et al. 2012 [38]	✗	Recruitment from multiple locations to promote respondent convenience	Identified barrier was that participants generally did not travel far to participate Difficult to obtain the trust of potential seeds in a short time
Vial et al. 2014 [18]	✗	Staff partnered with community organizations and local stores frequented by target population	21.9% of participants who completed the survey were excluded: approximately half of these did not meet inclusion criteria and others had missing data
Wylie & Jolly 2013 [19]	✗	Multiple methods for seed selection improved access to target group	Seed selection significantly influenced which subgroups within a population were accessed
Bogic et al. 2012 [27] ^G^	✓ 22 months	Interviews conducted at multiple sites	Authors suggest that the difficulty in recruiting a representative sample of refugees was linked to the absence of detailed population data in the target countries. The lack of registry data in the UK (compared to Italy and Germany) resulted in variation in recruitment methods across countries, which may have led to non-representative samples
Dunlavy 2001 [31] ^G-Thesis^	Not reported	Local cultural, community and political organizations assisted with recruitment Interviews conducted in locations convenient to participants Translators available to assist with survey administration	Snowballing methodology naturally excluded those not connected with the social networks targeted in the study

Long recruitment periods were identified in four studies to facilitate recruitment from hidden group. Non-peer-reviewed publications are emphasize in bold in table

^G^ Identified in grey literature search. ^G-Report^ Government reports identified in grey literature search. ^G-Thesis^ Dissertations identified in grey literature search

## Discussion

The reviewed studies demonstrate that it is possible to achieve a representative sample in RAS groups using either (or both) probability or non-probability sampling techniques, if the following requirements are met: a) engaging the target group, and b) key research design considerations. Both of these elements are discussed in reference to examples from the 20 studies in this review. We will also discuss issues reported in these 20 studies regarding the barriers to representative sampling, and suggest strategies for overcoming these barriers.

### Engagement with the target group

Engagement with the target group was universally identified as necessary for creating a representative community sample of hidden groups, including RAS. Engagement strategies included developing culturally responsive translated materials [23–26, 28, 30–35, 37, 39, 40], ongoing active engagement with target community members and leaders [24, 32, 35, 40], field-workers who spoke the language [23, 30, 32, 33, 35, 37, 39, 40] or were members of the target community [24, 32, 35], recruitment and site visits after hours and weekends [24, 40], and conducting the research at multiple sites to address travel limitations [27, 38].

### Key research design considerations

All reviewed studies identified research design considerations essential in developing representative community samples of RAS. More than half of the studies (60.0%) reported an a priori aim to recruit a representative sample [19, 23, 28, 30, 31, 33, 35–40]. This aim clearly articulated the study intent and guided the study design.

A second key study design consideration was the establishment (or identification) of a reliable sampling frame for the hidden group, necessary for both representative sampling and to assess sample representativeness. Overall, 15 (75.0%) of the studies reported a sampling frame [23–31, 33–37, 39] and nine of these had access to government resettlement databases/registries [23, 26, 27, 29, 30, 33, 34, 36, 37]. Given that governments in major countries of resettlement maintain resettlement records, when used in conjunction with ethical and transparent recruitment methods, a reliable sample frame can be developed in collaboration with government bodies.

In the absence of a readily available sampling frame for the target population, some studies reported creative methods to construct a suitable frame. These methods fell into two types of frames: 1) creating lists of names and details for every member of the target group [24, 25, 32], and 2) obtaining non-identifiable data describing demographics and areas of residence [18, 35, 38, 40]. The first frame had the advantage of allowing a random sample to be drawn from the list [24, 25, 32]. Both frames enabled the representativeness of the sample to be confirmed. A good example of the first approach is a Canadian study rated in this review as having high level evidence for a representative sample that had created a comprehensive sample frame by identifying 4854 households with at least one Ethiopian refugee resident [32]. This resource intensive study included methods to identify and confirm potential Ethiopian names from telephone books. It also described the importance of developing strong community networks with the target group to facilitate participation. The result of this 25-month study stage was a list of almost all Ethiopian refugees residing in the city of Toronto. An example of the second approach was a study involving 1165 Somalian and Oromo refugees in the United States of America (USA) in which sample demographics were compared with available demographics from public records of school enrolments, birth statistics and state resettlement records [40] to determine representativeness.

In some research, the lack of representativeness in a sample is addressed by statistical techniques such as weighting [42], a conventional design feature used in survey research involving a statistical analysis plan. Weighting methods were reported in five studies [25, 30, 37–39]. One Australian study [37] weighted responses by both demographic characteristics of the underlying population taken from governmental settlement records, and by sampling rates, which differed between strata in the sample. Used together, these weighting techniques produced a high-quality dataset, broadly representative of the sample frame targeted in the study [37].

Recording the number and characteristics of people who refuse to participate in research is an important form of data collection for all study types [20, 43, 44], as it can inform the researcher about the degree of non-representativeness in a sample and the potential for selection bias [17]. However, in many studies, only the response rate is reported and even then some response rates may be inaccurate. The majority of reviewed studies (75.0%) reported response rates [23–28, 30, 32–37, 39, 40] ranging between 41% [28] and 99% [35]. The high response rates reported in three studies, 95% [36], 97% [40], and 99% [35], may cast some doubt about accuracy, because it is uncommon to have nearly perfect rates of recruitment. When collected ethically, information about non-respondents can provide a greater understanding of the overall sample and sample frame. For example, in a large multiphase epidemiologic study of prevalence of exposure to torture in Somalian and Oromo refugees in the USA, records of 35 people who refused participation were collected to assess selection bias [40].

The use of multiple non-probability sampling methods was shown to be effective in producing representative samples. Although devising and implementing diverse sampling strategies may require additional resources, it appeared to enhance sample representativeness by facilitating access to diverse social networks within the target group. For example, in the previously mentioned American study of 1165 Somalian and Oromo refugees, participants were recruited by cluster sampling (41%), social networking (21%), snowball (31%) and convenience sampling (7%) [40]. Snowball sampling was often used in studies to reach those not readily accessible, e.g., recently arrived migrants and extremely isolated people [25, 27, 31, 32, 35, 40]. Having multiple and diverse ‘seed’ snowball or linkage starting points was recommended so that people could be accessed from different social networks [19, 35, 38, 40]. In the study of refugee migrants in China, 12 individuals of varied age, gender, occupation, and residential address were recruited as initial ‘seeds’ [38].

RDS is a non-probability sampling method that was designed for sampling hidden groups [19, 38, 45, 46]. It involves identifying seeds who then recruit usually between 0–3 participants; participation and recruitment are often incentivised. An advantage of RDS is that participant social networks can be mapped, as seeds are given individually coded ‘coupons’, which they then pass onto those that they recruit [19, 45]. The coupons are returned to the researcher when the recruited participant presents to takes part in the research. RDS was intended as a means of generating unbiased population estimates, but samples can vary considerably depending on initial seed selection, resulting in unstable outputs and reduced representativeness [47]. Evidence of this instability was seen in one of the studies that used RDS, where two seed groups were established for the purposes of comparing the influence of different methods of seed selection on recruitment. Results indicated that initial seed selection had the potential to strongly influence the type of participants; where one of the seed groups under investigation tended to recruit participants similar to themselves, the opposite was true of the second seed group [19]. As with all non-probability sampling, RDS has several strengths, it is a cost-effective method of recruitment, particularly from hidden groups. RDS also enables the recording and understanding of participants’ social networks. Although this can assist in analysis, it cannot guarantee representative sample compositions.

When possible, sampling strategies should also include a probability (random) sampling component to promote sample representativeness. Probability sampling procedures were used in almost half (45.0%) of the reviewed studies [23–28]. There were four studies that applied some type of probability sampling, supplemented with non-probability sampling such as snowballing or convenience sampling [23–25, 27]. For example, randomly selecting participants from a large pool of primary care registries was likely to over-estimate the prevalence of mental illness among Somali refugees in the UK, therefore the study included additional community sampling [24]. There may be concern regarding the representativeness of the study sample when both probability and non-probability sampling methods are used [27]. However, the use of only non-probability sampling in hidden groups can potentially draw out certain types of individuals needed for attaining representativeness [45].

Another strategy to improve sampling, if resources are available, is to invite all eligible participants from within the defined sample frame. Three studies adopted this approach; two of these were large studies involving Australian refugees that were rated high for a representative sample [30, 37]; and the third, rated medium, was a Swiss study that sampled consecutively from a national register of adult asylum seekers [34].

Long recruitment periods of between 12 and 25 months facilitated inclusion of refugee groups in four studies based in United Kingdom [24], Germany [27], Canada [32], and the USA [40]. The previously mentioned Canadian study that identified 4854 households with at least one member from the target group was resource intensive; sampling took over two years [32]. However, engagement with the target community is a time consuming process. A study of refugee migrants in China reported significant difficulties in obtaining the trust of potential seed participants in a short time, regardless of how the study was presented. Even among persons who initially appeared interested, some failed to attend the study appointment and subsequently dropped out of the study. This resulted in modifying the recruitment strategies and engaging seeds who already had established relationships with the researchers [38].

Online social network sites are new and potentially extensive sampling frames that can be used to target groups over a wide geographical area; for example, Facebook is a social-network website with more than 1.2 billion active users worldwide [18]. The effectiveness of social networking sites to recruit from hidden populations was examined in one study, which, together with field recruitment, used Facebook and dating websites to generate a sample of 3640 men who have sex with men [18] The study reported that in addition to being cost effective, Facebook offers particularly powerful new targeting capabilities that researchers may be able to exploit to gain access to hidden groups. This online recruitment method may be applicable to certain RAS sub-groups, for example, technologically literate refugees. However, it is noted that online recruitment methods may not be useful in all RAS groups due to barriers to Internet use, lack of technological literacy, safety concerns, and accessibility issues [48].

### Barriers and limitations to creating a representative sample from a hidden group of refugees and asylum seekers

Barriers and limitations were identified in creating a representative sample from a hidden group of RAS. These included the use of the popular snowballing technique which by design, cannot produce a probability sample of observations (and therefore no weights), since there is no way of determining the number of persons who ‘know’ each person in the sample. Barriers in engaging the target group for research included fear of breach of confidentiality. For example, in the previously discussed study with Somalian and Oromo refugees in the USA, trust between the researchers and community was reported to be important, as participants needed reassurance that their research involvement was confidential and would not jeopardize their public credibility [40]. Postal surveys were also viewed as a barrier to representative samples because many members of RAS groups might not be able or disinclined to respond. For example, in a study of refugees living in Sweden, a 65-question survey on mental health was mailed to 413 households yielding a 63% response rate; this response to a postal survey could be considered acceptable, but the study reported that non-responders in this case were likely to be biased towards those with poor health, therefore limiting representativeness of the mental health results obtained [26]. This same study also commented on another barrier that was addressed in their study design, namely to reduce focus on ethnicity in the survey and cover letter in order to engage refugees who no longer self-identified as refugees [26]. The utility of using census data to identify the target group was recognised by one study as a limitation, as census data is not always accurate for locating small, mobile refugee migrants and illegal migrants [25]. Geographic information systems were used to assess representativeness in yet another study and showed that despite the aim to recruit a diverse sample of migrant workers in China, the majority of participants resided or worked in close proximity to the study sites, therefore limiting the generalizability of the result to populations outside of these areas [38].

### Study limitations and strengths

During the process of conducting this review, we encountered an unanticipated result: half of the publications identified from the grey search were in fact peer-reviewed publications (4/8, 50%) that were not detected in the main search. One was a thesis [31], which explains why it was not identified in the main search. Another publication by Ao et al. [23] was only identified via an item describing the same study found in the grey literature [41], a likely explanation being that the Ao et al. [23] paper was only published very recently. The remaining two peer-reviewed papers were not identified in the main search, as their titles, abstracts, keywords and Medical Subject Headings did not include key terms used in our search strategy despite describing relevant concepts. This finding provides evidence for the potentially inconsistent indexing of such publications, a previously documented limitation of the literature on RAS [49]. It also raises the possibility that other relevant articles were not identified by this review.

A further limitation was the exclusion of non-English publications. Although the grey literature search strategy was designed to identify relevant research from countries with the highest proportional intakes of RAS groups [22], including many non-English speaking countries, we were unable to obtain and translate studies in languages other than English due to time and resource constraints. The possibility of language bias can therefore not be ruled out. In addition, the majority of studies included in the review were conducted in high-income countries that actively accept refugees for resettlement and are not likely to be generalizable to low- and middle-income countries where most refugees live [22]. Finally, the inclusion of studies rated as having *medium* level evidence of obtaining a representative sample may limit conclusive comments regarding the effectiveness of some of the strategies discussed in this review.

The inclusion of study designs other than gold-standard randomized controlled trials was a limitation but also a strength of this review; the descriptive studies, case-studies and studies using non-probability sampling techniques provided insights into ways to increase representative sampling with hidden groups. However, the degree of heterogeneity between studies meant that results could not be combined statistically in a meta-analysis.

A strength of this review was the identification and inclusion of non-peer reviewed but highly relevant governmental reports [28, 29, 37] and a protocol article [30], found in the grey search. This shows the importance of including grey literature when investigating RAS groups, as governments are often well placed to undertake studies with these populations and the resulting reports are not always published in academic forums. We plan to detail our grey search in a separate ‘how-to’ publication so that other researchers can use this effective technique to search for relevant literature about RAS groups.

## Conclusion

This review suggests that representative samples of RAS and other hidden groups residing in the community can be generated, but that generating such samples requires specific efforts, including actively engaging the populations of interest, and incorporating the careful use of non-probability sampling, as well as other design considerations. In summary, key design considerations revealed in the reviewed studies were: an a priori aim to recruit a representative sample; a reliable sampling frame to check sample representativeness; recording of response rates and non-responder characteristics depending on ethical considerations; the requirement for long recruitment periods; the use of multiple non-probability sampling methods, including snowballing to access the most isolated; the use of multiple and diverse seed starting points to access different social networks; the tracking of respondent network recruitment; and, when possible, the inclusion of a probability sampling component. Finally, online social networking sites are providing new forms of sampling frames that potentially enable access to hidden groups across large geographical ranges. We anticipate that the findings from this study will assist researchers aiming to recruit representative samples of RAS groups, and will also encourage more rigorous reporting of future studies so that the representativeness of samples of RAS groups in research can be more readily assessed.

## Additional files

Additional file 1: Appendix A. contains the list of countries that were focussed on in the grey search. (DOCX 13 kb)
Additional file 2: Appendix B. contains the flow diagram for the main search. (DOC 75 kb)
Additional file 3: Appendix C. contains the flow diagram for the grey search. (DOC 69 kb)
