# Supplementary material for: A systematic review of studies with a representative sample of refugees and asylum seekers living in the community for participation in mental health research
Source: BMC Med Res Methodol. 2017 Mar 2;17:37. doi: 10.1186/s12874-017-0312-x (PMC5335792; doi:10.1186/s12874-017-0312-x)
Supplement: Additional file 1: Appendix A. — contains the list of countries that were focussed on in the grey search. (DOCX 13 kb) [file 12874_2017_312_MOESM1_ESM.docx]

**Additional file 1**

**Appendix A. The top 20 countries in the UNHCR global rankings in 2010 of highest refugee “third country resettlement” intake per 1000 inhabitants plus Australia, Canada and United States of America.** Technically according to UNHCR, the first country is where the refugees come from, the second country is where they spill into after fleeing, and the third country is where they resettle^1^. A systematic search of grey literature centred on these 23 countries.

| **Ranking for ‘Refugees to 1,000 inhabitants”** | **Country** |
| --- | --- |
| 1 | Jordan |
| 2 | Syrian Arab Rep. |
| 3 | Congo, Rep. of |
| 4 | Chad |
| 5 | Montenegro |
| 6 | Djibouti |
| 7 | Malta |
| 8 | Iran (Islamic Rep. of) |
| 9 | Pakistan |
| 10 | Kenya |
| 11 | Sweden |
| 12 | Ecuador |
| 13 | Norway |
| 14 | Yemen |
| 15 | Mauritania |
| 16 | Serbia |
| 17 | Germany |
| 18 | Venezuela (Boliv. Rep. of) |
| 19 | Luxembourg |
| 20 | Switzerland |
| ------------------- | ------------------- |
| 28 | Canada |
| 69 | Australia |
| 73 | USA |

^1^ United Nations High Commissioner for Refugees. UNHCR Global Trends Report - Table 24. Indicators of host country capacity and contributions 2010. https://docs.google.com/spreadsheets/d/1bJR5428WlSjYVCZ5WmSiapDkj9D9nHXGrjstc4KzzWY/edit?hl=en_US&pref=2&pli=1#gid=11. Accessed July 2 2015
