# Supplementary material for: A systematic review of studies with a representative sample of refugees and asylum seekers living in the community for participation in mental health research
Source: BMC Med Res Methodol. 2017 Mar 2;17:37. doi: 10.1186/s12874-017-0312-x (PMC5335792; doi:10.1186/s12874-017-0312-x)
Supplement: Additional file 2: Appendix B. — contains the flow diagram for the main search. (DOC 75 kb) [file 12874_2017_312_MOESM2_ESM.doc]

**Additional file 2**

**Appendix B. Flow Diagram of main search strategy to identify eligible papers.** Asylum-seeker (AS).

*Screening*

*Identification*

*Eligibility*

*Included*

Records identified through database searching

(n = 1,857)

Additional records identified through reference checks

(n = 29)

Records after duplicates removed

(n = 893)

Title/abstracts screened

(n = 893)

Records excluded

(n = 655)

Full-text articles assessed for eligibility

(n = 238)

Full-text articles assessed for representative samples

(n = 36)

Articles excluded (n = 203)

Non-community sample &/or service focus (n = 24)

Non-health study (n = 17)

Not primary article (n = 5)

Not hidden group (n = 3)

No recruitment (n = 3)

Sampling method insufficiently detailed or not transferable to other groups (n = 151)

Sex workers (n = 18)

Men who have sex with men (n = 33)

HIV (n = 30)

Drug users (n = 40)

Other (n = 30)

Included papers (n = 12)

Refugee and AS (n = 9)

General-transferable: primary papers that describe a methodology transferable to sampling RAS (n = 3)

Migrants (n = 1)

Men who have sex with men (n = 2)

MAIN SEARCH

**Included**
