# Supplementary material for: A systematic review of studies with a representative sample of refugees and asylum seekers living in the community for participation in mental health research
Source: BMC Med Res Methodol. 2017 Mar 2;17:37. doi: 10.1186/s12874-017-0312-x (PMC5335792; doi:10.1186/s12874-017-0312-x)
Supplement: Additional file 3: Appendix C. — contains the flow diagram for the grey search. (DOC 69 kb) [file 12874_2017_312_MOESM3_ESM.doc]

**Additional file 3**

**Appendix C. Flow Diagram of grey search strategy to identify eligible papers.** Asylum-seeker (AS).

**Included**

Records identified by Internet search

(n = 92)

*Screening*

*Identification*

Records after duplicates removed

(n = 87)

Title/abstracts screened

(n = 87)

Records excluded

(n = 35)

Full-text articles assessed for eligibility

(n = 52)

Full-text articles assessed for representative samples

(n = 21)

Articles excluded (n = 31)

Non-community sample &/or service focus (n = 10)

Non-health study (n = 1)

Not primary article (n = 0)

Not hidden group (n = 4)

No recruitment (n = 11)

Sampling method insufficiently detailed or not transferable to other groups (n = 0)

Not adults (n = 6)

*Eligibility*

*Included*

Included papers (n = 8)

Refugee and AS (n = 8)

GREY SEARCH
